# Discovery and management of accessory hemidiaphragm during video-assisted thoracoscopic segmentectomy for pulmonary nodule: a case report

**DOI:** 10.3389/fsurg.2026.1790499

**Published:** 2026-03-26

**Authors:** Yijun Fan, Tingrui Mei, Lei Liu, Xiaoling Gu, Ya Gou, Lin Jiang, Jingsi Dong

**Affiliations:** 1 West China School of Medicine, Sichuan University, Chengdu, China; 2 Department of Thoracic Surgery, West China Hospital, Sichuan University, Chengdu, China; 3 Lung Cancer Center / Lung Cancer Institute, West China Hospital, Sichuan University, Chengdu, China; 4 West China Medical Technology Transfer Center, Chengdu, Sichuan, China

**Keywords:** accessory hemidiaphragm, case report, membranectomy, segmentectomy, video-assisted thoracoscopic surgery

## Abstract

An accessory hemidiaphragm is a rare congenital intrathoracic malformation characterized by a fibromuscular diaphragm located superior to the normal diaphragm, typically featuring a central hiatus through which pulmonary vessels and bronchi traverse. This anomaly is often associated with scimitar syndrome. We present a unique case of an isolated accessory hemidiaphragm—devoid of the characteristic vascular anomalies typical of scimitar syndrome—discovered incidentally during thoracoscopic surgery performed for a pulmonary nodule. This report elaborates the rationale for performing a concurrent partial membranectomy and underscores the necessity of an individualized surgical strategy in such cases.

## Introduction

An accessory hemidiaphragm is an exceptionally rare congenital anomaly, with only about 40 cases reported worldwide. This report details a unique case discovered incidentally during uniportal VATS segmentectomy for a pulmonary nodule. The distinctiveness lies in its isolated presentation—characterized by cardiac dextroposition but without the core anomalous pulmonary venous return of scimitar syndrome—and its proactive surgical management. Unlike conservative approaches, a concurrent total membranectomy was performed. This was a prospective, functionally-driven decision to eliminate the membrane's mechanical barrier, thereby optimizing post-resection lung expansion and thoracic remodeling. This case highlights the necessity of individualized surgical strategies when such anomalies are encountered incidentally.

## Case presentation

**Figure 1:**
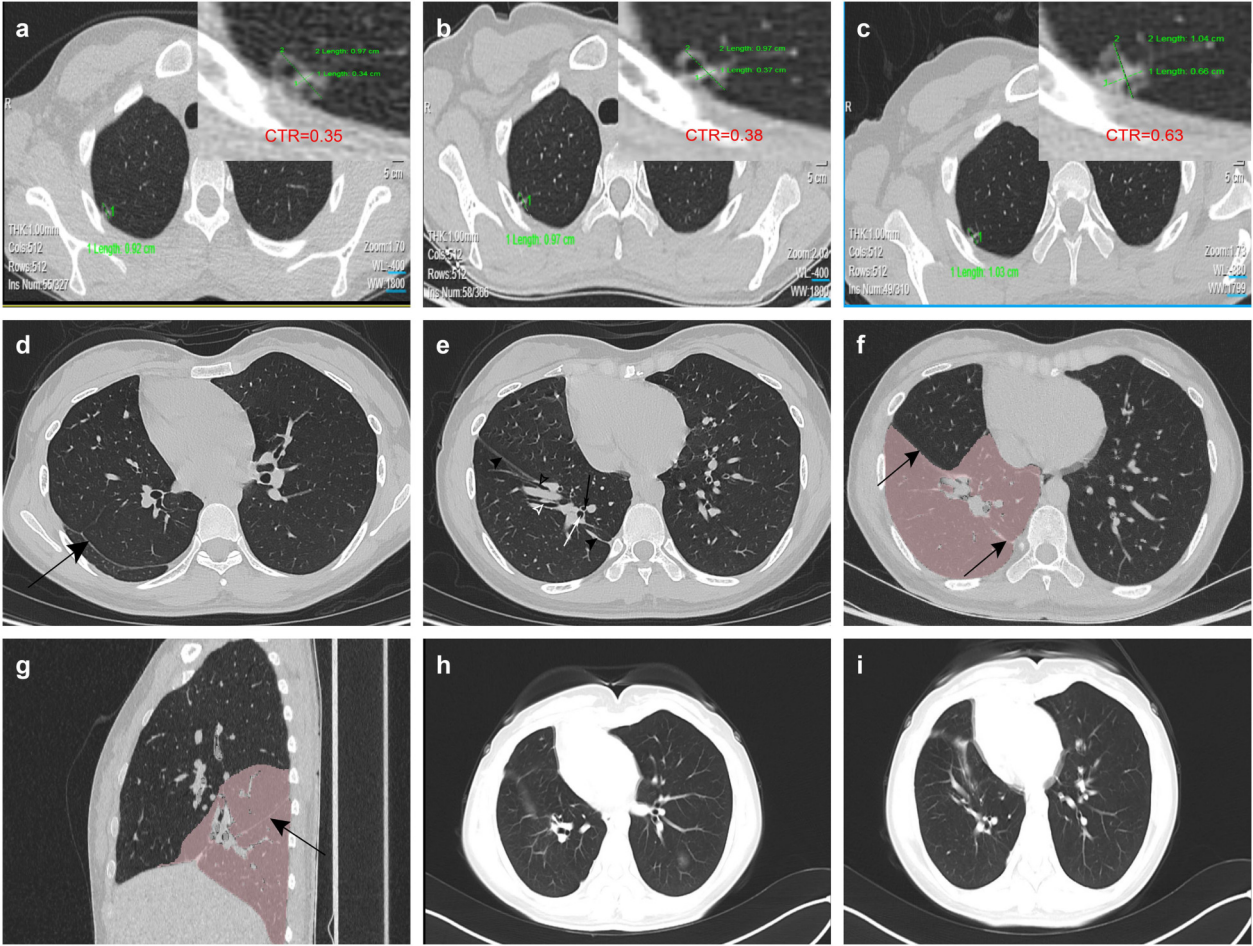
The diagnostic and therapeutic imaging workflow: preoperative nodule follow-up, identification of accessory hemidiaphragm, and postoperative assessment. **(a–c)** Serial preoperative follow-up chest CT images of the pulmonary nodule in the right lung. **(a)** January 2025, CTR=0.35 **(b)** April 2025, CTR=0.38 **(c)** October 2025, CTR=0.63. **(d, e)** Preoperative axial chest CT images illustrating the accessory hemidiaphragm. **(d)** The black arrow highlights the accessory hemidiaphragm appearing as a continuous linear high-density structure. **(e)** Detailed view of the hiatus. The black arrowhead indicates the accessory hemidiaphragm. Key structures traversing the hiatus are annotated: black hollow arrowhead, medial basal segmental bronchus (B7); white hollow arrowhead, anterior basal segmental bronchus (B8); black long arrow, posterior basal segmental bronchus (B10); white long arrow, lateral basal segmental bronchus (B9). **(f,g)** Preoperative CT images with three-dimensional segmentation. **(f)** Sagittal view. The black arrow traces the accessory hemidiaphragm, showing its anterior attachment to the diaphragmatic pleura and posterior attachment to the pleura at the 6th/7th intercostal space. **(g)** Axial view. The black arrow indicates the accessory hemidiaphragm. The red area outlines the hypoplastic right lower lobe as segmented by the software. **(h, i)** Postoperative axial chest CT images at 1-month follow-up. Both images demonstrate well-expanded residual lung tissue, satisfactory thoracic cavity remodeling, and absence of significant atelectasis or inflammatory changes.

A 27-year-old female presented with complaints of “occasional chest tightness and decreased exercise tolerance.” Preoperative computed tomography (CT) revealed a pure ground-glass nodule located in the apical segment of the right upper lobe. Upon follow-up, the nodule had evolved into a mixed ground-glass nodule with a maximum diameter of approximately 1.0 cm (Figures 1a–c). The consolidation-to-tumor ratio (CTR) had increased from 0.35 at initial detection to 0.63 on the most recent CT scan prior to surgery (Figures 1a–c). One month prior to surgery, a CT scan was repeated following a 7-day course of oral moxifloxacin, which showed persistence of the nodule. The nodule was located adjacent to the pleural surface, posing a potential risk of pleural dissemination if malignant (1). In addition, the patient experienced significant anxiety and strongly requested surgical resection. Considering that the nodule could be easily localized intraoperatively, the surgical risk was low, and the planned resection would result in minimal loss of pulmonary function, surgical resection was recommended after thorough discussion with the patient.

**Figure 2:**
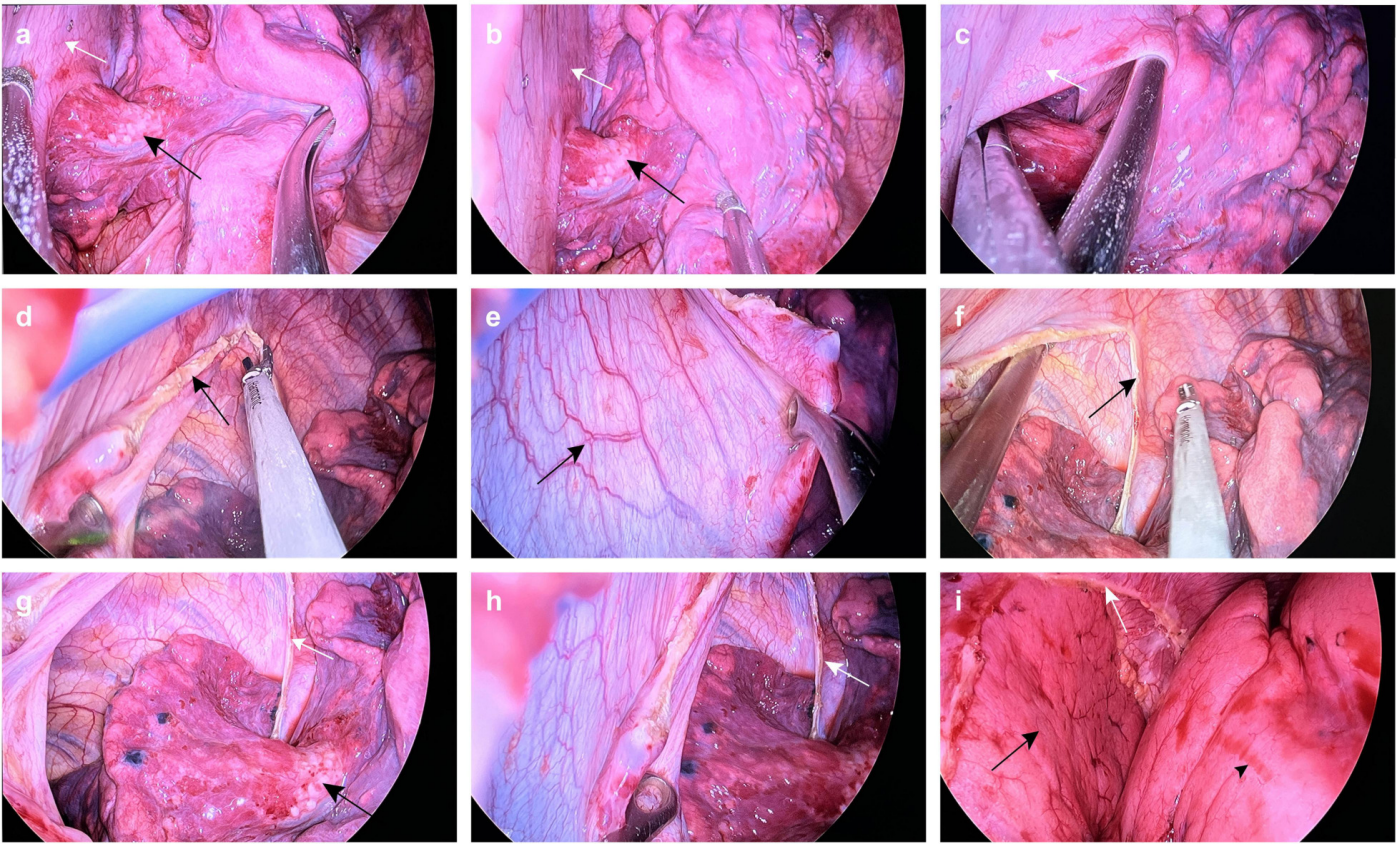
Intraoperative findings and surgical management of the accessory hemidiaphragm. **(a–c)** Before intervention. **(a, b)** Intraoperative views showing the accessory hemidiaphragm (white arrows). Black arrows indicate clusters of micronodular whitish patches on the adjacent lung parenchyma near the membranous attachment. **(c)** Dissection of the accessory hemidiaphragm to expose the underlying normal lung tissue, revealing its elastic and resilient membranous nature. **(d–f)** During resection. **(d)** Incision of the membrane using surgical instruments; the black arrow marks the cutting edge. **(e)** The membrane after detachment from one side of its attachment (black arrow). **(f)** The cut edge of the membrane (black arrow) remains at a distance from the chest wall, minimizing the risk of vascular injury and bleeding. **(g–i)** After resection. **(g)** The resection margin of the membrane (white arrow) and adjacent clustered whitish patches (black arrow). **(h)** View of the resection margin (white arrow). **(i)** Residual membrane after partial resection (white arrow). The black long arrow indicates the right lower lobe, and the black arrowhead points to the right middle lobe.

**Figure 3:**
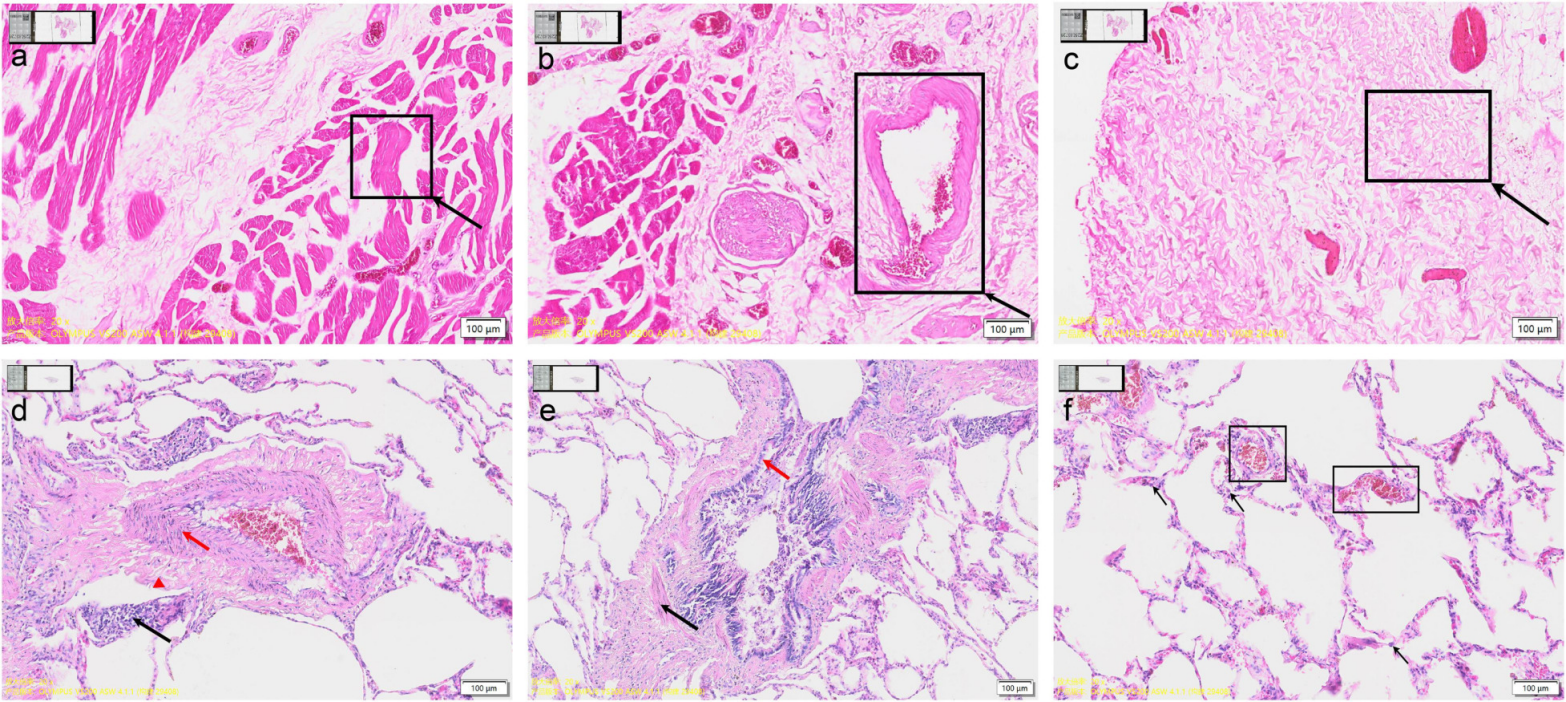
Pathological findings of the abnormal membrane and the lung nodule. **(a–c)** Abnormal membrane: **(a)** Smooth muscle tissue. **(b)** Vascular structures within the membrane. **(c)** Well-arranged fibrous tissue. **(d–f)** Postoperative pathology of lung nodule, consistent with chronic inflammation: **(d)** Cholesterol granuloma. The red arrow indicates epithelioid cells; the black arrow indicates infiltrating inflammatory cells; the red triangle marks fibrous tissue. Slit-like cholesterol clefts are present within the lesion. **(e)** Bronchiole. The red arrow points to ciliated columnar epithelium; the black arrow indicates the circular smooth muscle layer. Inflammatory exudate is visible within the lumen. **(f)** The boxed area shows congested alveolar septal vessels. The black arrow indicates focal alveolar epithelial hyperplasia.

The patient underwent a uniportal video-assisted thoracoscopic surgery (VATS) with a planned right upper lobe apical segmentectomy. Intraoperative exploration confirmed the presence of the nodule. The most significant finding was an anomalous membrane completely separating the right lower lobe, through which the right lower lobe artery, vein, and bronchus traversed. Furthermore, multiple clusters of micronodular whitish patches were observed in the lung parenchyma adjacent to the membranous attachment, consistent with subpleural fibroplasia induced by chronic mechanical friction (Figure 2). After apical segment resection of the lung, the abnormal membranous tissue was completely resected using an ultrasonic scalpel, fully exposing the right lower lobe. The procedure was completed successfully. Postoperatively, the patient received patient-controlled analgesia (PCA) with hydromorphone hydrochloride combined with ondansetron. The recovery was smooth: the chest drain was removed on postoperative day 2, and the patient was discharged on day 4. Postoperative pathology confirmed the pulmonary lesion to be a chronic inflammatory process, and the accessory membrane was identified as a fibromuscular structure (Figure 3).

A postoperative review of the preoperative CT images clearly delineated the membrane. In the left sagittal CT view, the membrane was displayed as a complete linear structure extending anteroinferiorly to posterosuperiorly, with its anterior end attaching to the diaphragmatic pleura and its posterior end attaching to the pleura at the level of the 6th/7th intercostal space. Axial CT images demonstrated the vascular structures of the right lower lobe traversing the membrane, and obvious cardiac dextroposition was also noted (Figures 1d–g).

## Discussion

The accessory hemidiaphragm typically features a central defect through which vital structures such as the pulmonary artery, pulmonary veins, and bronchi pass (2). The incidence of accessory hemidiaphragm is extremely low; according to a review by Radhakrishnan et al., approximately 40 confirmed cases were reported globally by 2014, with only 9 cases in adults (3). The clinical spectrum is broad, ranging from complete absence of symptoms to recurrent respiratory infections and respiratory distress due to lung compression, pulmonary hypoplasia, or associated cardiovascular anomalies (e.g., scimitar syndrome) (4). Beyond the accessory hemidiaphragm, our patient was also found to have dextroposition of the heart and mild hypoplasia of the right lung; however, they lacked the anomalous pulmonary venous return, aberrant systemic arterial supply, and other core features characteristic of classic scimitar syndrome (5). Therefore, we classified this case as an “isolated accessory hemidiaphragm”.

The distinctive feature of this case lies in the proactive management of the accessory hemidiaphragm during the segmentectomy performed for the pulmonary nodule. Current literature suggests that asymptomatic cases can be observed (3). Iijima et al., in a case involving pulmonary metastasis, proposed that incision at the membranous hiatus during concurrent thoracic surgery is sufficient to prevent complications such as lung herniation or vascular compression (6).

**Figure 4:**
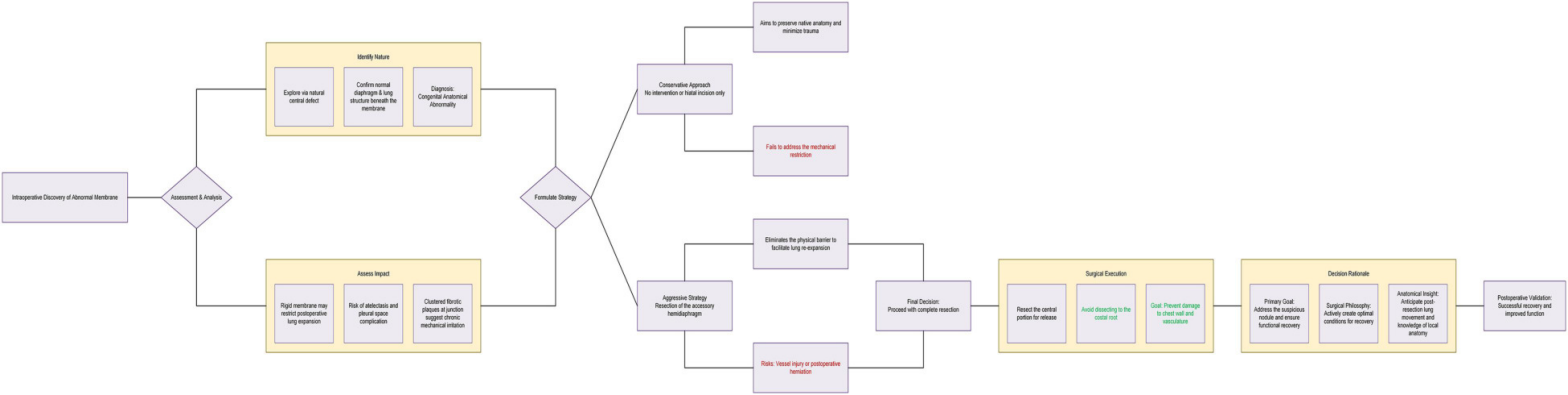
A management mind map for accessory hemidiaphragm encountered during radical resection of lung cancer.

In our patient, however, the planned right upper lobe apical segmentectomy would lead to upward shift and re-expansion of the remaining middle and lower lobes. Leaving the intact accessory membrane in place would have created a rigid mechanical barrier, severely limiting postoperative lung expansion and thoracic remodeling, thereby increasing the risk of atelectasis and impaired functional recovery. Consequently, we proactively performed a whole membranectomy. This decision was not due to intraoperative obstruction but was a prospective measure to optimize functional outcome (Figure 4).

Postoperatively, the patient reported marked alleviation of preoperative chest tightness and exertional dyspnea. Postoperative pulmonary function testing showed a mild decline compared to preoperative values, which was considered acceptable given that the patient was still in the early recovery phase (Table 1). Follow-up imaging confirmed well-expanded residual lung tissue and satisfactory thoracic configuration without significant atelectasis (Figures 1h, i). Taken together, the clinical symptoms, imaging findings, and pulmonary function assessment support the functional benefit of the combined procedure.

**Table 1:** Perioperative changes in pulmonary function.

Pulmonary function index	Preoperative	Postoperative (1 month)
FVC [L]	2.97	2.83
FEV1 [L]	2.54	2.36
FEV1%	78.3%	74.6%
MVV [L/min]	101.6	94.4
MVV%	87.8%	82.8%
DLCO [mmol/min/kPa]	9.43	8.15
DLCO%	99.9%	88%

## Conclusion

We report a rare case of an isolated accessory hemidiaphragm discovered during segmentectomy for a pulmonary nodule. The particularity of this case lies in the proactive partial membranectomy performed to optimize post-resection lung expansion and thoracic remodeling. This case also highlights the diagnostic challenges associated with this anomaly: despite performing three-dimensional reconstruction and multi-planar analysis of CT images, including axial, sagittal, and coronal views, no characteristic radiographic features were identified that could precisely delineate the abnormal membrane. Only subtle findings suggestive of fibrotic thickening of the interlobar fissure were observed. This underscores the difficulty in definitively identifying an accessory hemidiaphragm using current imaging techniques. For patients with an accessory hemidiaphragm found during surgery for other thoracic conditions, surgeons should comprehensively evaluate its mechanical impact on postoperative physiology and formulate an individualized management plan to achieve optimal functional recovery. For accessory hemidiaphragmatic malformation incidentally detected intraoperatively during thoracic surgery, concomitant resection of the accessory hemidiaphragm is recommended to avoid impairing pulmonary re-expansion and secondary remodeling of intrathoracic organs.

## Data Availability

The original contributions presented in the study are included in the article/Supplementary Material, further inquiries can be directed to the corresponding author.
